# Low Prevalence of Active Tuberculosis among High-Risk Pregnant and Postpartum Women in Sweden: A Retrospective Epidemiological Cohort Study Using and Evaluating TST as Screening Method

**DOI:** 10.1155/2018/3153250

**Published:** 2018-07-18

**Authors:** Maria Bullarbo, Martina Barnisin, Nina Vukas Radulovic, Åsa Mellgren

**Affiliations:** ^1^Department of Obstetrics and Gynecology, Sahlgrenska Academy, University of Gothenburg, Gothenburg, Sweden; ^2^Department of Gynecology, Närhälsan, Mölndal, Sweden; ^3^Women's Clinic, Södra Älvsborgs Hospital, Borås, Sweden; ^4^Department of Gynecology, Kungsbacka Hospital, Kungsbacka, Sweden; ^5^Clinic of Infectious Diseases, Södra Älvsborgs Hospital, Borås, Sweden

## Abstract

**Objective:**

Studies on the prevalence of active tuberculosis (TB) and latent tuberculosis infection (LTBI) among high-risk pregnant and postpartum women are few and prevalence is not well known. The methods used for diagnosing and treating TB and LTBI also differ both within and between countries. The aim of the study was to investigate the prevalence of TB and LTBI among high-risk pregnant and postpartum women in a Western Region of Sweden using tuberculin skin test (TST) as screening method. Secondary aims were to evaluate the effectiveness of the screening method and possible negative labour and neonatal outcomes among TST-positive women.

**Methods:**

Pregnant women attending an antenatal care unit (ACU) allocated for TST screening were investigated and followed up for two years postpartum.

**Results:**

Only one woman out of 902 screened women in the study group was diagnosed with active TB because of TB symptoms and not because of positive TST. 36% of the skin-tested women fulfilled criteria for LTBI. No difference in perinatal outcome was found between women with and without positive TST.

**Conclusions:**

Our findings suggest that TST screening of high-risk women may not be an effective strategy, since the prevalence of active TB is low. Investigating pregnant and postpartum women with TB symptoms instead of TST for screening could be an option in low TB prevalence areas. The criteria for diagnosing and treating LTBI should be clearly stated.

## 1. Introduction

Tuberculosis is globally a major medical health problem, and according to WHO Global Health Observatory data from 2016, TB is the ninth leading cause of death worldwide and the leading cause from a single infectious agent, being ranked above HIV/AIDS [1]. In 2016, an estimated 10.4 million people were diagnosed with active TB, and there were 1.3 million TB-caused deaths among HIV-negative people and an additional 374 000 deaths among HIV-positive people [1]. TB incidence is higher in men than in women, with 90% of the cases being adults and 10% being children [2]. The exact global prevalence of TB in pregnant women is not defined, but incidence is expected to be as high as in general population. A study from UK reported an incidence of 4.2 per 100.000 maternities [3], and a study from US found prevalence of 7,1 TB cases per 100.000 pregnancy-related hospitalizations [4].

There are studies from the pretreatment era showing that pregnancy does not predispose women to progressive TB [5, 6]. According to results from earlier reports, progression rate was only 2% among pregnant and postpartum women who had received TB chemotherapy, with most occurring after pregnancy [6]. TB incidence has also been shown to be significantly higher up to 180 days postpartum but not during pregnancy [7]. However, pregnancy itself may confound the symptoms of early TB, which may delay diagnosis and treatment. Among pregnant women being screened for and diagnosed with TB, the majority were in fact asymptomatic [8, 9]. As a consequence, unawareness of TB among pregnant women may lead to congenital infection in the newborn child, although this is a rare condition even in high endemic areas [10]. Active TB is also associated with a higher risk of pregnancy-related complications and in-hospital death [4].

In high endemic areas, maternal TB is associated with increased risk of small-for-gestational age, preterm, and low-birth-weight infants and high perinatal mortality [11]. According to WHO, TB among mothers is associated with a 6-fold increase in perinatal deaths and a 13-fold risk of preterm labour and low birth weight [12]. Whether latent TB infection (LTBI) affects perinatal outcome is not well studied.

Screening for TB among pregnant women in Sweden was first recommended in 2012 by the National Board of Health and Welfare (NBHW) if they had an origin from a TB high-risk country according to WHO or had been exposed to TB disease [13]. The incidence of TB in pregnant women in Sweden is low but it is not well defined or investigated. Screening methods in Sweden have by tradition differed regionally, some of which have used chest X-ray (CXR) for screening and some tuberculin skin test (TST) or IGRA test [14]. TST, most commonly performed with purified protein derivate (PPD), was previously considered the test of choice for diagnosing TB in pregnant women. The strength with TST screening is that it is associated with good compliance rates and it is an effective way to identify high prevalence of latent TB, although not active disease [15]. In recent years, blood tests have been developed which measure T-cell gamma interferon release in response to specific tuberculin antigens (IGRA, interferon-gamma release assay), but there are still rather few studies performed in pregnant women. Because of IGRA being an expensive method, it has until recently been suggested that TST should be preferred as first screening method [16, 17]. Although pregnant women have suppressed cell-mediated immunity to tuberculin according to in vitro studies [18, 19], this does not appear to be clinically relevant [20], and pregnancy is not believed to alter the response to a TST. But up to 1/4 of immunocompetent persons with active TB, pregnant or not, have a false negative test result [21].

As there is no gold-standard method, screening methods still differ within the country, and TST as a screening method should therefore be investigated. It is also of interest to evaluate if a TB screening program is of enough value in areas with low prevalence of TB.

## 2. Objective

The aim of the study was to evaluate the result, the value, and effectiveness of TST screening for active and latent TB infection among high-risk pregnant women at an antenatal care unit (ACU) in Sweden, with a follow-up for two years after labour. Secondary outcomes were to compare labour and fetal outcomes between women with negative and positive PPD in the TST.

## 3. Materials and Methods

This is a retrospective epidemiological cohort study of a prospective TB screening program launched in 2008. From November 2008 to June 2012, all pregnant women attending the ACU at Borås municipality, in the Western part of Sweden, were assessed for TST screening. Inclusion criteria were either origin from a high-risk country according to WHO (incidence of TB ≥ 100/100000 inhabitants) or having been in contact with suspected TB disease. For the TST 0,1 ml tuberculin PPD RT 23 SSI, 2 TU was used and the test was performed by a trained nurse at the ACU. The tuberculin was injected intracutaneously, and the diameter of the local induration of the skin was measured after 48-72 hours. The TST was performed at any time of gestational age but preferably in the first trimester pregnancy. A diameter of the induration of ≥10 mm was considered as positive PPD and further investigation with CXR was recommended. In cases of a diameter of ≥20 mm, the women were referred to the clinic of infectious diseases (CID) for clinical investigation including CXR and/or IGRA blood test.

All pregnant women with positive PPD were followed up during the screening period and up to two years postpartum (the last included followed up until 2015). For diagnosing active TB among the study group, medical records were used at the ACU and the CID. As follow-up, controlling for women diagnosed with TB two years postpartum, registers from the Department of Communicable Disease Control and Prevention, Region Västra Götaland, and the Public Health Agency of Sweden (PHAS) were used (all cases of active TB are reported to PHAS according to regulations in the communicable diseases act in Sweden). High-risk women who declined to be skin-tested or did not show up for CXR or clinical investigation were also included in the follow-up procedure in order to find postpartum women diagnosed with active TB.

Women with positive PPD (the TST used) were compared with women with negative PPD for labour and fetal outcomes. Data regarding results from CXR, baseline data, and labour and fetal outcomes were collected from medical records at the ACU and labour ward. The collected parameters were maternal age, nationality, parity, body mass index (BMI), history of smoking, gestational age, delivery mode, hours of active labour, pH in the arterial umbilical cord, birth weight, fetal Apgar score at five minutes, and need for treatment at a neonatal intensive care unit (NICU).

### 3.1. Ethical Approval

The study was approved on 2013-05-06 (Dnr 3012-13) by the Regional Ethical Committee, Gothenburg, Sweden.

### 3.2. Statistics

For continuous variables, mean (SD)/median (min; max)/n= is presented. For comparison between groups, Fisher's exact test (lowest 1-sided p-value multiplied by 2) was used for dichotomous variables, the Mantel-Haenszel Chi Square test was used for ordered categorical variables, and the Mann–Whitney U test was used for continuous variables. A p-value < 0,05 was considered statistically significant.

## 4. Results

**Figure 1:**
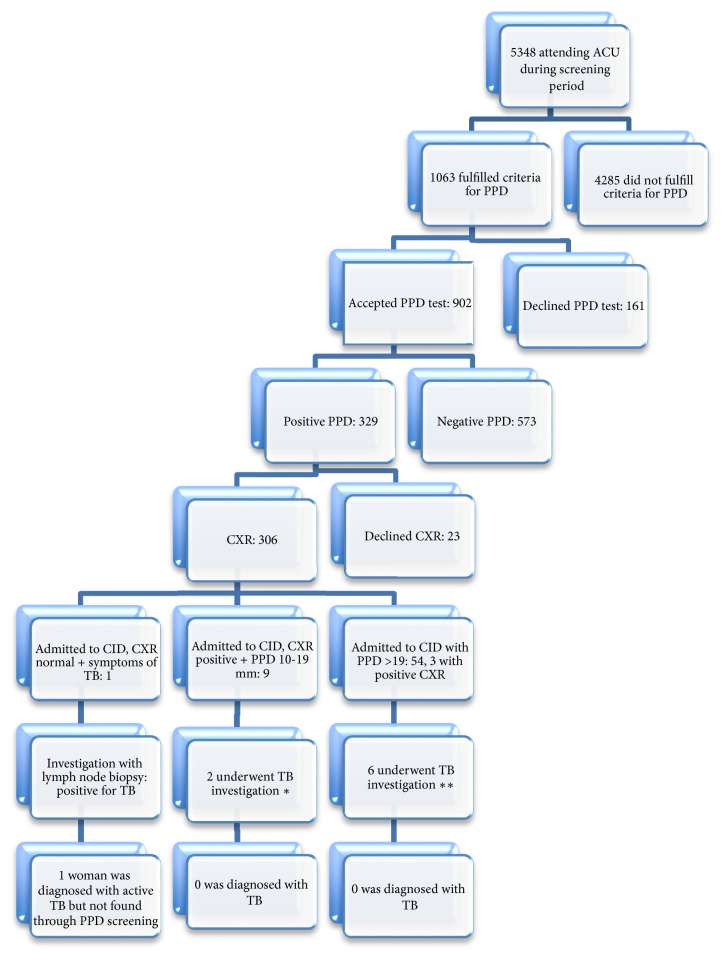
Flowchart describing TST of pregnant women (using PPD) at Borås ACU during November 2008–June 2012. *∗* represents 1 CT scan of thorax and 1 IGRA negative. *∗∗* represents 1 IGRA positive, 3 urinary TB culture, 1 urinary TB culture + culture from nasopharynx, and 1 CT scan of thorax.

The results of the TST screening program are illustrated in Figure 1. Of a total of 5348 pregnant women being registered at the ACU in Borås during the study period, 20% of the women fulfilled the criteria for TB screening, but 15% of these declined TST. None in the declining group was diagnosed with active TB during pregnancy or 2 years postpartum. Thus, 902 pregnant women were skin-tested, among which 36% were tested PPD-positive. Of these, 34% underwent CXR investigation, whereas 7% declined. Eleven women had TB suspected changes on the CXR investigation (4% of all with examined CXR). Seventy-five women had a PPD of at least 20 mm (22% of all positive PPD) of which 54 were admitted directly to the CID for further investigation including CXR. The remaining 21 women out of 75 declined further investigation or were not admitted to the CID by the midwife. In addition, nine women had a PPD between 10 and 19 (one with unclear PPD diameter) but had pathological signs on the CXR and were therefore also admitted to CID for further TB investigation. Finally, one woman that was screened with PPD 16 mm but with normal CXR was later in her pregnancy admitted from a general practitioner (GP) to the CID because of hemoptysis and a swollen lymph node. This woman was diagnosed with active TB and was the only woman in the TST screening program diagnosed with active TB.

Of the 64 patients admitted to CID, 35 women reported having been BCG-vaccinated and 20 were uncertain. Only 59 performed a CXR as some patients declined or did not show up at calling. Seven women underwent further TB investigation. No woman was given LTBI treatment.

There were no differences between the positive and negative PPD groups regarding baseline data (Table 1) and labour or fetal outcomes (Table 2).

When evaluating the study population for postpartum TB, 368 women had had a new pregnancy and revisited the ACU. None of them had been diagnosed with postpartum active TB, and none were found with active TB among the remaining 534 patients, formerly TST screened, when checked at the Department of Communicable Disease Control and Prevention, Region Västra Götaland, the CID, and PHAS. Nineteen women had migrated abroad with a possible shorter follow-up period. One was lost to follow-up due to secret identity.

## 5. Discussion

This is the largest Swedish study that evaluates TB screening in pregnant women, and we found that only one woman out of 902 TST screened pregnant women was diagnosed with active TB, and no vertical transmission occurred.

The result of a low incidence of active TB among pregnant women raises a question about the efficacy and benefit of a TST screening program at the ACU. The program requires intervention, duty time from midwives and two extra visits at the ACU for the pregnant women, to find one out of 902 pregnant women with active TB, a woman that alternatively could be admitted to the CID and diagnosed with TB because of symptoms. Thus, in the Swedish setting, the close follow-up by midwives and the information and awareness of TB symptoms among pregnant women and health care providers may be the most important instrument for early diagnosis and treatment.

Although TST screening is considered harmless and of low cost, it is associated with several weaknesses such as false positive and negative results, and a positive PPD cannot distinguish between active and latent TB. Also, internationally, the PPD limits measured in millimeter (mm) for positive or negative PPD have differed. CXR used for TB screening has also weaknesses mainly due to interreader variability [22]. However, screening with IGRA is becoming more common in Sweden as the rate of false positive results is lower compared to TST screening and has also been recommended by PHAS since 2017 [23]. The advantage of IGRA is that it eliminates cross reaction to similar mycobacterial infections and is not affected by earlier BCG vaccination. Of importance, however, is the fact that screening for TB during pregnancy has been shown to be complex, as results seem to depend not only on screening method (IGRA/TST) but also on gestational age or time point of the postpartum period [24].

The results from this study, with a cohort of women with an origin from high-prevalence countries, indicate prevalence of 36% or more of possible LTBI as there was a drop-out of 15%. This is in accordance with a smaller Swedish study, where 40% of the women had a positive PPD [14]. At the time of our study, the national recommendations were published in March 2012, and according to them, diagnosis of LTBI was to be set by individual evaluation of exposure, chest X-ray, PPD result, and other risk factors [13]. Sixty-four pregnant women were admitted to CID for further investigation, but none were given LTBI treatment. The new Swedish recommendations regarding LTBI published in 2017 have an algorithm but with no strict recommendations, leaving the decision to the clinician [23]. There is a lack of prospective data of safety of antepartum LTBI therapy regarding hepatitis and efficacy. Altogether, this raises the question about the advantages, cost-benefits, and risks. According to a systematic review investigating prevalence of LTBI among pregnant women, the prevalence differed between 14 and 48% [25]. Three of these studies compared IGRA with TST and found a concordance between the tests of 77, 88, and 91%. The authors also concluded that further studies are needed concerning antepartum LTBI treatment.

We found no negative outcome regarding labour or fetal outcomes for women being PPD-positive with a possible LTBI. The aim of the 2017 Swedish TB screening recommendation in pregnant women is to prevent serious congenital TB infection [23]. In this study, no child was congenitally infected with TB, and this condition is, as mentioned earlier, rare.

## 6. Conclusion

The main finding in this study is that active TB is rare in Sweden and is not detected with TST, and none diagnosed with LTBI received medical treatment. These findings suggest that TST screening of eligible pregnant women is not an effective strategy. More studies are needed regarding screening with other methods and safety with LTBI treatment, and there is a need for both validation and criteria for such treatment. Alternatively, the option is to give BCG vaccination to high-risk infants, focusing on TB investigation of pregnant women with TB symptoms.

## Figures and Tables

**Table 1:** Baseline data

Variable	PPD-positive ≥10 (n=327)	PPD-negative <10 (n=574)	**p-value**
**Maternal age**	28.1 (5.2)	27.8 (5.7)	0.41
	28.0 (16.0; 43.0)	27.0 (10.0; 45.0)	
	n=327	n=574	

Parity			

0	133 (40.7%)	265 (46.2%)	

1	102 (31.2%)	184 (32.1%)	

2	56 (17.1%)	81 (14.1%)	

3	18 (5.5%)	23 (4.0%)	

4	10 (3.1%)	10 (1.7%)	

5	4 (1.2%)	3 (0.5%)	

6	4 (1.2%)	5 (0.9%)	

7	0 (0.0%)	2 (0.3%)	

13	0 (0.0%)	1 (0.2%)	0.070

Proportion parity			

Nulliparity	133 (40.7%)	265 (46.2%)	

Multiparity	194 (59.3%)	309 (53.8%)	0.13

Smoking habits			

Nonsmoker	270 (82.6%)	484 (84.3%)	

Smoker	57 (17.4%)	90 (15.7%)	0.55

BMI	25.0 (4.9)	24.5 (4.6)	0.091
	24.0 (15.0; 47.0)	24.0 (16.0; 44.0)	
	n=321	n=561	

For categorical variables, n (%) is presented.

For continuous variables, mean (SD)/median (min; max)/n= is presented.

For comparison between groups, Fisher's exact test (lowest 1-sided p-value multiplied by 2) was used for dichotomous variables, the Mantel-Haenszel Chi Square test was used for ordered categorical variables, and the Mann–Whitney U test was used for continuous variables.

**Table 2:** Comparison of labour and fetal outcomes between pregnant women with PPD ≥10 and normal PPD <10.

Variable	PPD-Positive ≥10 (n=327)	PPD-Negative <10 (n=574)	p-value
Gestational length (weeks + days)	39.8 (1.8)	39.7 (1.8)	0.51
	40.0 (26.0; 42.7)	39.9 (27.0; 42.7)	
	n=321	n=565	

Delivery mode			

Emergency cesarean section	24 (7.5%)	49 (8.7%)	

Elective cesarean section	21 (6.5%)	23 (4.1%)	

Normal vaginal delivery	263 (81.7%)	459 (81.5%)	

Vacuum extraction	14 (4.3%)	32 (5.7%)	0.32

Duration in hours of active labour	4.87 (3.55)	5.07 (3.42)	0.30
	4.00 (1.00; 25.00)	4.00 (1.00; 26.00)	
	n=279	n=498	

Birth weight (grams)	3414 (518)	3395 (559)	0.44
	3440 (1375; 4884)	3398 (510; 4910)	
	n=319	n=564	

Apgar score after 5 minutes	9.83 (0.70)	9.81 (0.94)	0.85
	10.00 (2.00; 10.00)	10.00 (0.00; 10.00)	
	n=318	n=561	

Apgar score after 5 min (0,1,2-10)			

0	0 (0.0%)	4 (0.7%)	

1	0 (0.0%)	0 (0.0%)	

2-10	318 (100.0%)	557 (99.3%)	0.13

Need for NICU			

No	263 (82.4%)	480 (85.3%)	

Yes	56 (17.6%)	83 (14.7%)	0.31

Arterial pH in the umbilical cord	7.27 (0.07)	7.27 (0.08)	0.90
	7.28 (7.03; 7.46)	7.28 (6.64; 7.43)	
	n=308	n=531	

For categorical variables, n (%) is presented.

For continuous variables, mean (SD)/median (min; max)/n= is presented.

For comparison between groups, Fisher's exact test (lowest 1-sided p-value multiplied by 2) was used for dichotomous variables, the Mantel-Haenszel Chi Square test was used for ordered categorical variables, and the Mann–Whitney U test was used for continuous variables.

NICU: neonatal intensive care unit.

## Data Availability

Data are available as an Excel file upon request from the corresponding author.
